# Editorial: New technologies for detection, monitoring and treatment of Parkinson's disease, volume II

**DOI:** 10.3389/fnagi.2023.1235265

**Published:** 2023-07-04

**Authors:** Maryam S. Mirian, Soojin Lee, Martin J. McKeown

**Affiliations:** ^1^Pacific Parkinson's Research Centre, University of British Columbia, Vancouver, BC, Canada; ^2^Faculty of Medicine (Neurology), University of British Columbia, Vancouver, BC, Canada

**Keywords:** Parkinson's disease, gait impairment, imaging-derived biomarkers, disease monitoring, disease detection

Parkinson's disease (PD) is currently diagnosed and monitored using clinical criteria. There is huge potential for new technologies to improve early detection, ongoing assessment, and understanding of the pathophysiology of PD to inform new therapies. This is the basis for this Topic, *New technologies for the detection, monitoring, and treatment of Parkinson's disease*. PD is a heterogeneous disorder affecting different brain and non-brain systems with varying rates of progression. One manifestation of disease progression is gait impairment, a significant feature that affects the quality of life of many individuals. Biomarkers, which can quantitatively monitor disease status and progression, and assess the effects of new treatments, are actively being sought. In this special issue, we have a set of five interesting accepted papers addressing gait impairment and imaging-derived biomarkers in PD.

In Joza et al. the authors examine the role of the pedunculopontine nucleus (PPN) in gait control. The study suggests that abnormalities in PPN connectivity, particularly in the right hemisphere involving regions such as the caudate nucleus and amygdala, contribute to gait dysfunction in PD. This finding highlights the importance of investigating overall brain networks and connectivity patterns to understand the neural basis of gait impairment. In addition, Guo et al. provide an overview of gait analysis as a tool for both detecting and monitoring PD. Gait impairments can serve as clinical signs for early PD detection and can be objectively measured, allowing for pervasive monitoring of patients in daily life. The paper discusses various gait analysis systems and automatic recognition methods, highlighting the importance of personalized interventions and smart devices for improving gait performance in PD.

The three other papers on this Topic describe novel brain imaging-based biomarkers for PD. Two papers emphasize the importance of incorporating subregions of the basal ganglia into the analysis. Pan et al. introduces a method for detecting PD using PET imaging. By analyzing dopaminergic activity in different subregions of the striatum, Pan et al. found that specific standardized uptake value ratios (SUVRs) had high diagnostic accuracy for PD. Liu et al. focuses on the diagnostic value of 18F-FP-DTBZ PET imaging for PD. This study demonstrates that the posterior dorsal putamen (PDP) can effectively differentiate PD patients from healthy controls. The last paper Shih et al. highlights the use of diffusion tensor imaging (DTI) to study white matter alterations in PD. DTI enables the detection of early axonal changes in PD, which may play a critical role in the disease's pathophysiology. The review emphasizes the significance of DTI in differentiating PD subtypes, understanding the progression of the disease, and optimizing treatment strategies such as deep brain stimulation (DBS).

In summary, these papers collectively contribute to the broader understanding of gait impairment in PD and its underlying brain imaging mechanisms. They highlight the potential of advanced imaging techniques, gait analysis systems, and DTI in diagnosing and monitoring PD, as well as guiding personalized interventions. The findings have implications for improving the quality of life for individuals with PD and advancing research in the field of neurodegenerative disorders.

It is important to consider some weaknesses, limitations, and potential biases that may affect the interpretation of the results of the accepted papers.

- Sample Size: all the studies have relatively small sample sizes, which may limit the generalizability of the findings. This is likely due to the relatively large expense associated with imaging studies. Larger-scale studies involving more diverse populations would be valuable to confirm and strengthen the observed correlations and diagnostic accuracy.
- Cross-sectional Design: The majority of the mentioned studies adopt a cross-sectional design, which captures a snapshot of the participant's condition at a specific point in time. Longitudinal studies that track individuals over time would provide more insights into the progression and predictive value of the observed biomarkers and connectivity patterns.
- Heterogeneity of Parkinson's disease: Parkinson's disease is a complex and heterogeneous disorder with various subtypes and clinical manifestations. The studies mentioned may not capture the full spectrum of PD, potentially limiting the generalizability of the findings to all PD patients.

Overall this Topic has exemplified the importance of new technologies in detecting and monitoring PD, with the ultimate goal of improving the lives of people living with the disease.

## Author contributions

MSM, SL, and MJM summarized the research papers included in the special issue, discussed the limitations, and potential biases inherent in the research papers. All authors contributed to the article and approved the submitted version.

